# Molecular Diffusion of ABCA1 at the Cell Surface of Living Cells Assessed by svFCS

**DOI:** 10.3390/membranes11070498

**Published:** 2021-06-30

**Authors:** Olga Raducka-Jaszul, Karolina Wójtowicz, Aleksander F. Sikorski, Giovanna Chimini, Yannick Hamon, Tomasz Trombik

**Affiliations:** 1 Department of Cytobiochemistry, Faculty of Biotechnology, University of Wrocław, Joliot-Curie 14a, 50-383 Wrocław, Poland; olgaraducka@gmail.com; 2 Department of Biotransformation, Faculty of Biotechnology, University of Wrocław, Joliot-Curie 14a, 50-383 Wrocław, Poland; karolina.wojtowicz@uwr.edu.pl; 3 Research and Development Center, Regional Specialist Hospital, Kamieńskiego 73a, 51-154 Wroclaw, Poland; sikorski.aleksander@wssk.wroc.pl; 4 Aix Marseille University, CNRS, INSERM, CIML, 13007 Marseille, France; chimini@ciml.univ-mrs.fr (G.C.); hamon@ciml.univ-mrs.fr (Y.H.); 5 Department of Biophysics, Faculty of Biotechnology, University of Wrocław, Joliot-Curie 14a, 50-383 Wrocław, Poland

**Keywords:** svFCS, ABCA1, nanodomains, molecular confinement, amphotericin B, ApoA1

## Abstract

Extensive studies showed the crucial role of ATP binding cassette (ABC) transporter ABCA1 in organizing the lipid microenvironment at the plasma membrane (PM) of living cells. However, the exact role of this protein in terms of lipid redistribution and lateral reorganization of the PM is still being discussed. Here, we took advantage of the spot variation fluorescence correlation spectroscopy (svFCS) to investigate the molecular dynamics of the ABCA1 expressed at the PM of Chinese hamster ovary cells (CHO-K1). We confirmed that this protein is strongly confined into the raft nanodomains. Next, in agreement with our previous observations, we showed that amphotericin B does not affect the diffusion properties of an active ABCA1 in contrary to inactive mutant ABCA1MM. We also evidenced that ApoA1 influences the molecular diffusion properties of ABCA1. Finally, we showed that the molecular confinement of ABCA1 depends on the cholesterol content in the PM, but presumably, this is not the only factor responsible for that. We concluded that the molecular dynamics of ABCA1 strongly depends on its activity and the PM composition. We hypothesize that other factors than lipids (i.e., proteins) are responsible for the strong confinement of ABCA1 in PM nanodomains which possibility has to be elucidated.

## 1. Introduction

Extensive research on the structure and function of the plasma membrane (PM) led to the development of the fluid mosaic model in 1972 by Singer and Nicolson. This model describes the plasma membrane as homogenous two-dimensional lipid bilayers in which proteins are placed [1]. In addition, further researches of Nicolson on the fluid mosaic model drew attention to the possible role of the extracellular matrix and the cytoskeleton in the diffusion of the molecules in the PM [2]. Other studies enriched the membrane model with the possible capability of the lipids to segregation between liquid-ordered and liquid disordered domains [3,4] and cholesterol ability to increase the PM lateral heterogeneity [5]. In 2006, a definition of lipid rafts (reviewed in [6,7]) was formulated and presents them as heterogeneous, highly dynamic, sphingolipid- and cholesterol-enriched domains (10–200 nm) that have the potential to form larger platform through the protein-protein and lipid-protein interactions [6,7,8] Several proteins including ABC transporters are known to be involved in the maintaining of the PM organization (recently reviewed in [9]). Among them, ABCA1 seems to play a major role in those processes.

ABCA1 promotes high-density lipoprotein (HDL) formation, cellular cholesterol efflux, and reverse cholesterol transport (RCT) [10,11,12]. The ABCA1 loss of function leads to the development of the Tangier disease, in which impaired RCT and accumulation of lipids have been observed [13]. Although the 3D structure of this transporter has been recently resolved [14], the mechanism of cholesterol efflux control is still being discussed. It has been reported that ABCA1 elicits an outward flip of phosphatidylcholine and negatively charged phosphatidylserine at the PM [15,16]. In addition, FLIM experiments have provided the first evidence that ABCA1 redistributes cholesterol present in the lipid raft between smaller pools [17]. Further, it was evidenced that nascent HDL molecules originate from PM lipid rafts under the coordination of ABCA1 [18]. We have demonstrated recently that ABCA1-mediated cholesterol efflux from the cell PM protects the cells against amphotericin B [19]. Thus, ABCA1 appears as a key controller of the lipid microenvironments with direct effects on the general molecular organization of the PM and subsequent processes.

Since the advent of the lipid rafts concept, many new techniques and tools have been developed that enable the study of lateral membrane heterogeneity [20,21]. In the 70’s, a method called fluorescent correlation spectroscopy (FCS) was introduced [22]. This method is characterized by very high temporal resolution, little sample consumption, short analysis time, and most importantly measurement of living cells under physiological conditions [23,24]. One of the modifications of the FCS method is spot variation FCS (svFCS), where the diffusion time is measured as a function of the different sizes of the observation area (waist, ω^2^) and plotted as the so-called diffusion law. The projection of the diffusion law intercept with the time axis provides the *t_0_* value. When applied at the plasma membrane of living cells, it allows the identification of the possible major molecular confinement modes of diffusing molecules such as isolated nanodomains (*t*_0_ > 0), cytoskeleton meshwork (*t*_0_ < 0), or free diffusion (*t*_0_ = 0) [23,25].

In the present study, we took advantage of previously developed and validated CHO-K1 cell lines stably expressing either active ABCA1 or A1 (A1G cells) or non-active catalytic mutant ABCA1MM or MM (MMG cells), both, in fusion with enhanced GFP (eGFP) [19]. Using the svFCS, we assessed the confinement of the ABCA1 at the PM of living cells and factors that influence its molecular diffusions, such as amphotericin B or ApoA1.

## 2. Materials and Methods

### 2.1. Cells

A1G and MMG cells were cultured in Ham’s F-12 Nutrient Mix (Gibco) supplemented with 10% newborn calf serum (NBCS, Gibco), 100 U/mL penicillin (Gibco), 100 µg/mL streptomycin (Gibco), and 2 mM L-glutamine (Gibco) (further referred as a complete Ham’s F12 medium) with the addition of 150 µg/mL Zeocin (Invitrogen). Cells were cultured at 37 °C with 5% CO_2_ in a humidified atmosphere. A1G and MMG cells were periodically verified by flow cytometry regarding ABCA1 expression. All cells were grown in *Mycoplasma*-free conditions and tested every month.

### 2.2. Reagents

Amphotericin B, apolipoprotein A1, cholesterol oxidase, and methyl-β-cyclodextrin (MβCD) were purchased from Sigma Aldrich. Cholesterol was purchased from Northern Lipids Inc. (Burnaby, BC, Canada).

### 2.3. Preparation of the Complexes of Methyl-β-Cyclodextrin (MβCD):Cholesterol

7.6 mg of cholesterol was resuspended in 153 µL of chloroform:methanol (2:1) in a glass tube. The solution was mixed and dried under a nitrogen airstream. Then 256 mg of MβCD resuspended in 2 mL of water was added drop by drop to the cholesterol solution. The resulting MβCD:cholesterol (100 mM:10 mM) complexes suspension was sonicated for 20 min in RT in a water bath and was shaken overnight at 37 °C with 500 rpm. The day after MβCD:cholesterol was filtered through a 0.22 µm filter and stored at −80 °C.

### 2.4. Preparation of the Cells to the svFCS Measurement

A1G and MMG cell lines were cultivated in a complete Ham’s F12 medium supplemented with 150 µg/mL Zeocin. The 8-wells Lab-Tek Chamber Slides (Nunc) were precoated overnight with complete Ham’s F12 medium. Two days before the experiment, cells were seeded at 1 × 10^4^ cells/mL in the precoated Lab-Tek Chambers. On the day of the experiment, the cells were washed twice with HBSS buffer supplemented with 10 mM HEPES, pH 7.4 (Gibco) (HBSS-HEPES) before treatments.

Cells were incubated in the presence of 20 µg/mL AmB in HBSS-HEPES buffer for 20 min at 37 °C. Then cells were analyzed by svFCS in the presence of AmB at 37 °C. The measurements were carried out for a maximum of one hour and a half due to the preservation of cell viability.

Alternatively, ApoA1 was directly added to the well in the final concentration of 10 µg/mL and incubated for 4 h prior to svFCS analysis, which was then performed in the presence of ApoA1 at 37 °C.

For lipid-raft probe diffusion analysis, cells were labeled with fluorescent sphingomyelin—bodipy-SM (Invitrogen by Thermo Fisher Scientific, Waltham, MA, USA) immediately before the measurements. Briefly, cells were washed once with HBSS-HEPES buffer and incubated with 0.075 µM lipid/BSA complex in HBSS-HEPES buffer for 10 min at room temperature in the dark. After staining, cells were washed three times with HBSS-HEPES buffer, and svFCS analysis was performed at 37 °C. Cholesterol oxidase treatment was performed for 1 h at 1 µ/mL beforehand svFCS experiments. The svFCS measurements were performed in the presence of cholesterol oxidase (0.1 µ/mL) within max. 1 h at 37 °C.

Cholesteroldepleted cells were washed three times with HBSS-HEPES buffer and incubated for 30 min at 37 °C with MβCD:cholesterol resuspended in F12 media supplemented with delipidated FBS (MβCD:cholesterol complexes dilution 1:100; final concentration 1 mM:0.1 mM). The svFCS measurements were performed in the presence of 0.1 mM:0.01 mM MβCD:cholesterol complexes within 1 h at 37 °C.

### 2.5. svFCS Measurements

The svFCS measurements were performed on a homemade apparatus based on a fluorescence microscope Axiovert 200 M (Carl Zeiss, Oberkochen, Germany) with an excitation 488 nm argon-ion laser beam, as described in Mailfert et al. [25,26].

#### 2.5.1. svFCS Data Recording and Analysis

Prior to the measurements on the cells, the waist size was calibrated using rhodamine 6G solution as described in Mailfert et al. [25,26]. For the measurement of the living cells, the 488-laser beam power was adjusted to 3–4 µW. The GFP-expressed cells were identified under widefield fluorescence illumination. The low fluorescence signal cells were preferable as the ACF is inversely proportional to the number of diffusing fluorescent molecules within the confocal spot. High fluorescence signal results in flat ACFs, which are difficult to fit and thus correctly calculate the mean diffusion time (*t_d_*) and mean number of diffusing molecules (N). In the next step, the XY confocal image and z scan of the selected cells were performed. The separation between the top and bottom plasma membrane was achieved. The maximal fluorescent intensity of the top membrane was chosen for the acquisitions. The fluorescence fluctuations were collected during 20 runs of 5 s each. The measurements were carried out on 10 to 20 individual cells. For each waist, the ACFs from all individual runs were examined for the stability of the count rate during recording. Then, the average of the selected ACFs (typically between 7 and 15) was fitted with a 2D diffusion model that is used for membrane proteins. The diffusion law was constructed from measurements obtained in at least four different waist sizes.

#### 2.5.2. Statistical Analysis

The statistical analysis was performed using the GraphPad Prism 7 software package (GraphPad). Error bars presented in the figure data points represent standard error (SE).

## 3. Results

### 3.1. ABCA1 Protein Is Confined in the PM Nanodomains

The partitioning of ABCA1 into lipid raft domains is a long-lasting question. Debates originate from the different biochemical strategies to purify detergent-resistant membranes. The use of non-ionic detergent such as Triton X-100 at 4 °C consistently gave rise to the non-DRM association of ABCA1 partitioning. Conversely, ABCA1 was found in DRM obtained by milder solubilization (Brij 96) at 37 °C. Although non-physiological the latter approaches have shown consistency with more accurate biophysical approaches based on FCS [27,28,29] as demonstrated for the raftophilic molecule Thy-1.

**Figure 1:**
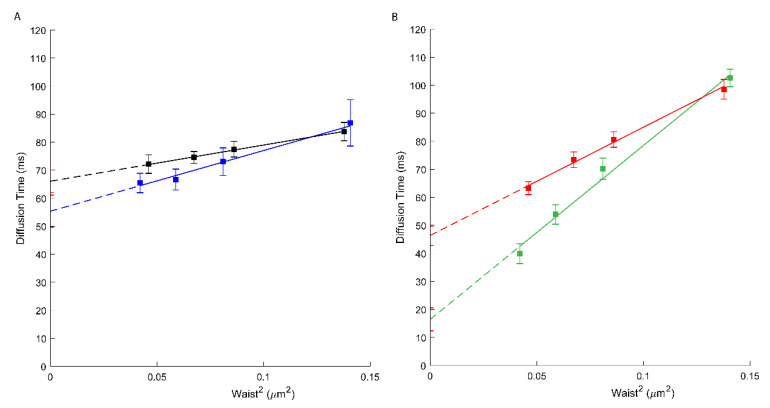
(**A**) svFCS diffusion laws for an active ABCA1 in non-treated A1G cells (black) and after amphotericin B treatment (blue). (**B**) svFCS diffusion laws for a non-active ABCA1MM in non-treated MMG cells (red) and after amphotericin B treatment (green).

We, therefore, have performed svFCS analysis to characterize the molecular diffusion of ABCA1 in A1G and MMG cells. As shown in Figure 1 and Table 1, the ABCA1 diffusion law is characterized by high positive *t*_0_ values, either for the WT or the mutated form of ABCA1 (respectively 66.06 ms; Figure 1A, black, and 46.39 ms; Figure 1B, red). A positive *t_0_* value is indicative of a dominant type of confinement of molecules dynamically trapped into isolated nanodomains [23]. Our results show the strong confinement of ABCA1 protein in the PM nanodomains with a significantly higher extent for an active ABCA1 in A1G cells. This indicates that the molecular diffusion of ABCA1 in the PM of living cells is at least in part dependent upon its activity. Surprisingly, the active ABCA1 was characterized by a much higher effective diffusion coefficient (*D_eff_* =1.94 µm^2^/s) than the non-active mutant in MMG cells (*D_eff_* = 0.65 µm^2^/s) (Table 1), which is fully unexpected for an integral membrane protein, and largely inconsistent with SPT based studies [30].

Contrary to the previously mentioned study, we did not observe a massive fraction of immobile molecules. As evidenced in Supplementary Figure S1, all count rates of our recording show oscillations around a constant median value. In the case of large amounts of immobile molecules, count rates would have been regularly decreasing across time, which is definitively not the case here. Additionally, the average number of diffusing molecules (N) per waist surface remains stable with a slight decrease at higher waists due to the lower illumination density as the laser power was kept constant for all waists (Supplementary Figure S2). Note that most of our recordings were performed on the upper membrane of the cells, reducing immobilization of molecules into adhesion foci, cytoskeleton pickets, or interaction with the extracellular matrix, which may have contributed to immobilize most of the ABCA1 molecules. Finally, the generated ACFs have relatively high amplitudes allowing their proper fit with the 2D diffusion model (Supplementary Figure S3).

### 3.2. Amphotericin B Does Not Impact the ABCA1 Molecular Confinement

In our recent publication [19], we demonstrated the possible role of ABCA1 in the interactions between the PM and amphotericin B (AmB). We proposed that in the presence of active ABCA1, AmB is captured in bulk cholesterol-AmB structures making AmB unable to penetrate the PM and cause cytotoxicity, unlike in cells expressing the loss-of-function mutant of ABCA1, MM [19].

Through svFCS analysis, we now show that AmB strongly affects the diffusion of the non-active ABCA1 in MMG cells (*t*_0_ drops to 16.43 ms; Figure 1B, green), whereas slightly affecting the diffusion of an active ABCA1 in A1G cells (*t*_0_ = 55.35 ms) (Figure 1A, blue; Table 1). It might be explained by the mechanism of action of AmB, which, from one side, may insert into the core of the PM but also may extract the lipids from the PM and disorganize the PM lateral organization. In A1G cells, the impact of AmB is low and possibly related to the fraction of cholesterol molecules released from the PM to AmB in ABCA1 dependent manner. In both cases, a decrease in the effective diffusion coefficient was observed (Table 1).

### 3.3. ApoA1 Affects the Diffusion Properties of ABCA1

**Figure 2:**
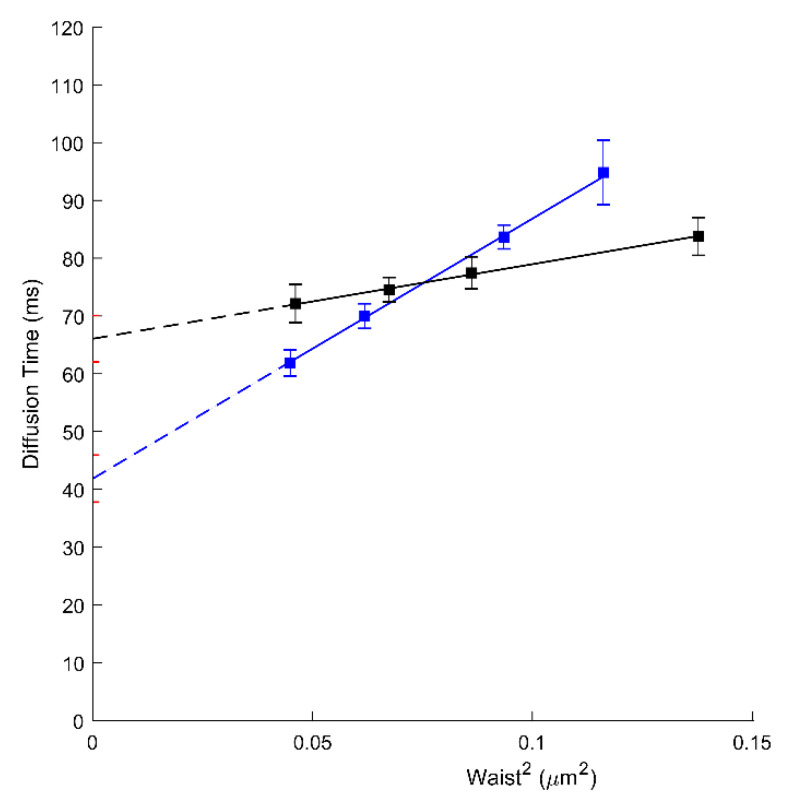
svFCS diffusion laws for an active ABCA1 in non-treated A1G cells (black) and after ApoA1 binding (blue).

Unless AmB is present, ApoA1 is a natural plasmatic acceptor of effluxed cholesterol and phospholipids in an ABCA1-dependent manner. It is still not clear, however, whether the docking of ApoA1 at the cell surface needs to physically interact with ABCA1 or requires an appropriate local PM environment upon ABCA1 activity [11,31,32]. Here, we assess by svFCS, how the ApoA1 influences the molecular diffusion parameters of ABCA1 protein. The svFCS measurements in the presence of ApoA1 showed an important change in the molecular diffusion of active ABCA1 in A1G cells but not in MMG cells. As shown in Figure 2, ApoA1 causes a significant decrease of the *t*_0_ value for the A1G (41.84 ms; blue) cells but does not considerably affect the *t*_0_ value for the MMG cells (Table 1). Moreover, the net change of the *t*_0_ value is followed by a significant decrease of the effective diffusion coefficient (*D_eff_* = 0.56 µm^2^/s) indicating that the presence of ApoA1 changes not only the surroundings of ABCA1 molecules but also slows down the diffusion rate of an active ABCA1. Since only the A1G cell line can bind ApoA1 and promote cholesterol efflux this observation was somehow expected. The efflux of cholesterol and other lipids in an ABCA1-dependent manner has a direct impact on the PM composition and organization. Moreover, the binding of ApoA1 itself impacts the physicochemical properties of the PM on one hand, and influences the dynamics of the ABCA1 transporter on the other, inducing weaker confinement of ABCA1 and its lower mobility.

### 3.4. Cholesterol as One of the Factors Influencing the Molecular Confinement of ABCA1

**Figure 3:**
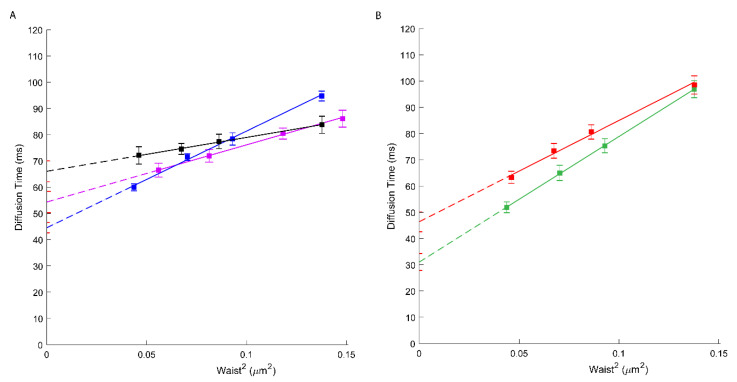
(**A**) svFCS diffusion laws for an active ABCA1 in non-treated A1G cells (black), after cholesterol oxidase treatment (blue) and after reload with cholesterol-methy-β-cyclodextrin (violet). (**B**) svFCS diffusion laws for a non-active ABCA1MM in non-treated MMG cells (red) and after cholesterol oxidase treatment (green).

To evaluate the involvement of cholesterol on ABCA1 molecular diffusion and confinement, we treated A1G and MMG cells with cholesterol oxidase (ChOx) prior to svFCS analysis. Cholesterol oxidase, by oxidation of cellular cholesterol from the outer leaflet of the PM, leads to the partial destruction of the lipid raft nanodomains and thus influences the PM lateral organization [33,34]. First, we evaluated the ability of ChOx to affect the lipid raft nanodomains by using a lipid-raft probe—fluorescently labeled sphingomyelin (bodipy-SM). As shown in Supplementary Figure S4, this fluorescent analog of SM displays a positive *t*_0_ = 14.5 ms value sensitive to the lowering of cholesterol. Upon ChOx treatment, this probe displays a *t*_0_ value close to zero (*t*_0_ = 1.15 ms) indicative of free-like diffusion. Similarly, the *D_eff_* value drops from 1.14 to 0.78 µm^2^/s upon ChOx treatment. In A1G and MMG cells treated with ChOx, an important decrease in the *t*_0_ value (44.54 ms; Figure 3A, blue and 31.03 ms; Figure 3B, green, respectively) was observed (Table 1). Additionally, an important decrease in ABCA1 mobility was also observed (*D_eff_* = 0.68 µm^2^/s) (Table 1). Finally, we measured the molecular diffusion of ABCA1 in cells treated with ChOx oxidase and reloaded with cholesterol:methyl-β-cyclodextrin complexes for 30 min. We observed a partial recovery of the ABCA1 molecular diffusion parameters where the *t*_0_ value increases from 44.54 ms to 54.34 ms, indicating that reloading cholesterol partially restores the original ABCA1 confinement (Figure 3A, violet; Table 1). This indicates that indeed cholesterol contributes in part to the confinement of ABCA1 WT or its loss of function mutant but is not the only factor determining the molecular diffusion properties of ABCA1 in these cell lines since still a strong positive offset of the *t*_0_ value remains.

## 4. Discussion

In this work, we took advantage of the svFCS technology to evaluate the molecular diffusion of ABCA1 at the PM of living cells under physiological conditions. This technique is not only characterized by extremely high temporal resolution (from ns to s) but also allows determining the modes of molecular diffusion of a membrane molecule, whether it is free, dependent on membrane nanodomains, or actin meshwork [23]. Whether ABCA1 function and localization depend on the lipid rafts at the PM is still highly debated. Most previous studies rely on the biochemical analysis of the PM solubility in mild detergents. For example, Sano et al. showed that ABCA1 is distributed to the domains that are solubilized by Triton X-100 and Brij 96. They suggested that ABCA1 is localized in the non-raft PM domains rich in phosphatidylcholine (PC) and may disturb detergent-resistant lipid raft domains [35]. This observation was in agreement with one of our early studies showing that active ABCA1 is not partitioned into the lipid rafts but affects lateral organization and function of other PM proteins [17]. On the contrary, Yamauchi et al. described ABCA1 as a protein detectable in the PM fraction resistant to mild detergent Brij 96, which would indicate that ABCA1 prefers lipid rafts environment in PM [36].

Our first observation is that ABCA1 displays a high *t*_0_ value indicative of a strong confinement into PM nanodomains. Moreover, this confinement is stronger for the active ABCA1 (A1G cells) than for non-active catalytic mutant ABCA1MM (MMG cells). Positive *t*_0_ value related to a situation where molecules are diverted from free diffusion but dynamic partitioned into isolated PM nanodomains. The diffusion time as a function of the diameter of the confocal excitation spot (so-called diffusion law) expresses the degree of the confinement of a given molecule at the PM. The more the *t*_0_ deviates from 0 (where *t*_0_ being a projection of the diffusion law on a time axis), the stronger the dominant of the confinement is. Surprisingly, the active ABCA1 is characterized by a rapid diffusion with a very high effective diffusion coefficient (*D_eff_* = 1.94 µm^2^/s) in contrast to the non-active ABCA1 mutant. This was somehow unexpected since a similar *D_eff_* was just obtained for lipids or GPI-linked proteins [28,29]. Here, the *D_eff_* is calculated as a slope 1/(4*D_eff_*) of the diffusion law. However, it is very important to note that the expression of the *D_eff_* value depends on several parameters and most importantly on the partition coefficient α that is defined by the time the molecule spends within the domain over the total diffusion time of a given molecule and describes partially the properties of the domain [37]. Since we are not able to evaluate by biochemical studies the real value of the partition coefficient, we could not speculate over its contribution to the high value of the *D_eff_*. Nevertheless, it has been previously evidenced by fluorescence recovery after photobleaching (FRAP) that raft-associated proteins are not characterized by lower diffusion rates than non-raft ones [38].

Although both proteins display a strong positive *t_0_* value, we anticipate that the biochemical nature of those nanodomains and their organization is not strictly the same. Besides cholesterol as one of the components of these domains (discussed below), other interactors (such as cytoplasmic adapters) may contribute to stronger confinement of active ABCA1. Indeed, it was reported that some proteins might interact with ABCA1 via the N-terminal PDZ domain and stabilized ABCA1 at the PM [39,40]. Moreover, considering the large and highly glycosylated extracellular domains of ABCA1, we cannot exclude the possible interactions with extracellular proteins such as galectins which may contribute to the differential confinement of these two ABCA1 isoforms. This, however, has to be further investigated and experimentally proved.

Based on the diffusion simulations, we might speculate that this type of diffusion regime is consistent with a free-like diffusion in a more viscous meso-domain bigger than the observation confocal spot [37], which may reflect the role of ABCA1 as a lipid exporter creating the specific lipid environment suitable for ApoA1 binding. The large *t_0_* offset, as a consequence of a high apparent diffusion time, might be related in fact, to a slow microscopic diffusion coefficient inside the ABCA1 created domains. In fact, based on biochemical studies, it has been proposed that active ABCA1 may form larger mesodomains that function as platforms for cholesterol and lipids efflux [35]. The ABCA1 diffusion law is characterized by a low slope, and we can observe that the mean diffusion time does not increase importantly between the smallest and the largest svFCS confocal spot (waist). This relatively stable diffusion time over several spatial scales may reflect the ABCA1-confinement organization, which is not sensitive to the increasing size of the svFCS confocal spot. It may indicate that the ABCA1 molecules reside in a domain (mesodomain) larger than the greatest svFCS confocal spot. These ABCA1 molecules are characterized by very high diffusion rates but spatially restricted within the domain area. Our results are drastically different from previous studies showing, by single-particle tracking (SPT) analysis, that active ABCA1 is almost immobile unless ApoA1 is present [30]. In our experiment, we can rule out a massive batch of immobile molecules, since very little photobleaching was observed even for long recordings (100 s) (Supplementary Figure S1). It would normally occur under laser illumination if the ABCA1-eGFP molecules were immobile. In addition, no drastic effects on the average number of diffusive species were observed throughout our analysis (Supplementary Figure S2). A possible reason for that discrepancy may stand for the differences in experimental approaches. svFCS has an approx. 100-fold higher temporal resolution when compared to SPT and we can actually register the fluorescence fluctuation resulting from rapid diffusion of ABCA1 molecules within restricted space that cannot be measured with a 30–100 ms time resolution of SPT. SPT and FCS report comparable diffusion coefficients when acquisition frequencies are similar, which is not to be the case here. Other discrepancies that may be noted compared to our study is that murine ABCA1 can behave differently than human ABCA1. Stably expressing cells might be in a more homeostatic state than transient transfectants analyzed two days after transfection (putatively in an unstable and very low expression phase, the level of expression of ABCA1 being reported as very low (0.3 molecule/µm^2^) probably very insufficient to induce cholesterol efflux). Moreover, most of our recordings were performed on the upper membrane, and not on the membrane contacting the support, to minimize steric hindrance of the extracellular matrix or trapping in the vicinity of adhesion foci. This partially explains the differences but further investigations would be needed to reconcile both approaches.

In our recent study we demonstrated that an active ABCA1 in A1G cells can protect the cells from the cytotoxic effects of amphotericin B (AmB) [19]. Here we assessed how AmB affects the diffusion properties of both, active and non-active ABCA1. As expected, our results showed that AmB has a limited effect on the molecular diffusion of ABCA1 in A1G cells but strongly affects non-active ABCA1 in MMG cells (Figure 1B, green; Table 1). These results support the conclusions from our previous publication. In fact, in A1G cells, AmB is not able to penetrate the PM and forms bulk structures with cholesterol released outside the cell due to ABCA1 activity. Here we observe that the *t_0_* value for ABCA1 in A1G cells decreases slightly, and this is probably an effect of ABCA1-dependent efflux of a fraction of cholesterol to AmB. However, in the case of MMG cells, this decrease of *t*_0_ value is more than 60% (from 46.39 to 16.43 ms; Figure 1B) and indicates an important alteration in the PM organization due to the AmB incorporation into the PM core and disorganization of existing PM architecture.

We also analyzed the influence of ApoA1 on ABCA1 diffusion. Indeed, non-lipidated ApoA1 has been shown to interact with the cells expressing ABCA1 as the initial step of HDL biogenesis through ABCA1-mediated cholesterol and phospholipid efflux. This has been for more than 20 years nearly exclusively evidenced in vitro, on cells loaded with fluorescent or ^3^H-cholesterol and incubated with pure ApoA1. Our observation demonstrated that ApoA1 changes the molecular diffusion parameters of an active ABCA1 but not the mutant form. Additionally, ApoA1 binding does not impact the number of diffusing ABCA1 molecules (N) (Supplementary Figure S5). A significant decrease in the *t_0_* value (from 66.06 to 41.84 ms; Figure 2; Table 1) indicates a change in the lateral organization of the PM. This is probably due to the ABCA1-mediated cholesterol efflux from the PM into ApoA1. Indeed, in our previous study, we demonstrated that A1G cells promote the efflux of radiolabeled cholesterol to ApoA1 in contrast to MMG cells [19]. The decrease of PM cholesterol content will directly impact the lateral organization of the PM and the confinement of proteins associated with it. Additionally, ApoA1 causes a significant decrease in the ABCA1 diffusion rate (*D_eff_* drops from 1.94 to 0.56 µm^2^/s; Table 1). The most possible explanation would be that the ABCA1 molecules are engaged directly in the cholesterol efflux process towards ApoA1 which severely impacts their dynamics. We cannot also exclude the direct physical interactions between ABCA1 and ApoA1 that may impact the A1 diffusion rates. However, in the previous study using the fluorescence cross-correlation approach it was evidenced that ApoA1 and ABCA1 do not interact at the same temporal space [31]. Our observations are also concordant with the SPT-based study [30] in the way that ApoA1 induces an important change in the ABCA1 diffusion behavior. However, the differences in the experimental approaches used (SPT vs. svFCS), unable us to compare directly these two studies.

Taking into consideration the fact that cholesterol is a crucial component of the lipid raft nanodomains and seems to play an important role in ABCA1 confinement at the PM we analyzed how the partial depletion of cholesterol by cholesterol oxidase (ChOx) influences the ABCA1 at the PM. ChOx is widely used to disturb lipid-raft nanodomains, since cholestenone does not associate with sphingolipids on the outer leaflet of the PM. Interestingly both, active ABCA1 and non-active ABCA1MM were affected by the ChOx treatment. In both cases, an important decrease in the *t_0_* value was observed (Figure 3, Table 1), indicating that nanodomains’ disorganization by ChOx partially abrogates the confinement of these molecules. We also measured the molecular diffusion of ABCA1 in cells treated with ChOx oxidase and reloaded with cholesterol:methyl-β-cyclodextrin complexes for 30 min. (Figure 3A; Table 1). As expected, we observed a partial recovery of the ABCA1 molecular diffusion parameters indicate that reloading with cholesterol re-establish the PM organization and restores the original ABCA1 confinement. Since the *t_0_* value remains positive, it also demonstrates that the ABCA1 molecular diffusion at the PM depends on other, still-unknown factors such as interactions with other proteins within the PM or at its proximity as discussed above.

The last important observation is that the effective diffusion coefficient *D_eff_* for ABCA1MM seems to be stable over different tested conditions (Table 1) in contrast to active ABCA1 where its *D_eff_* changes significantly what might be interpreted as a hallmark of its activity independently in the changes of the *t*_0_ value which indicates the degree of the confinement of analyzed molecules.

## 5. Conclusions

Our studies using the svFCS approach demonstrated that ABCA1 diffusion properties at the PM depend on several factors and are directly related to the PM composition and ABCA1 activity. This protein is confined into the domains of the PM, of which cholesterol is a component but not the only one. In agreement with our recent results, we demonstrated that AmB affects the PM organization and diffusion parameters for only non-active ABCA1MM. Finally, ApoA1 binding influences the active ABCA1 molecular diffusion at the cell PM in relation to the cholesterol efflux process.

## Figures and Tables

**Table 1:** Summary of the diffusion parameters—t_0_ and D_eff_ for ABCA1 (A1) and ABCA1MM (MM).

Molecule (+ Treatment)	t_0_ [ms] ± SEM	Deff [µm^2^/s] ± SEM
A1	66.06 ± 4.02	1.94 ± 0.71
MM	46.39 ± 3.64	0.65 ± 0.07
A1 + AmB	55.35 ± 5.72	1.15 ± 0.45
MM + AmB	16.43 ± 4.09	0.4 ± 0.03
A1 + ApoA1	41.84 ± 4.06	0.56 ± 0.07
MM+ApoA1	45.96 ± 2.84	0.75 ± 0.08
A1 + ChOx	44.54 ± 1.95	0.68 ± 0.04
MM + ChOx	31.03 ± 3.24	0.52 ± 0.04
A1 + ChOx + MβCD:Chol	54.34 ± 4.09	0.86 ± 0.16

## Data Availability

Not applicable.

## Supplementary Materials

The following are available online at https://www.mdpi.com/article/10.3390/membranes11070498/s1. Figure S1: Representation of typical fluorescence fluctuation records from 100 s time frames for diffusion of ABCA1 in A1G cells (A), ABCA1MM in MMG cells (B) at different waist sizes (1: 0.15 µm^2^, 2: 0.21 µm^2^, 3: 0.27 µm^2^, 4: 0.43 µm^2^). A representation of fluorescence fluctuation record from 100 s time frame for the molecule which is photobleached is presented in C (waist size: 0.07 µm^2^); Figure S2: Evolution of ABCA1 molecule’s density (N/µm^2^) in the function of the waist surface in A1G and MMG cells. The differences are not statistically significant; Figure S3: Examples of the multiple ACF fit (lower panel in green) with 2D diffusion model for ABCA1 in A1G cells at different waist surfaces (A: 0.15 µm^2^, B: 0.21 µm^2^, C: 0.27 µm^2^, D: 0.43 µm^2^). The upper panel represents the deviation from the fit (in red); Figure S4: svFCS diffusion laws for a Bodipy-SM in non-treated CHO-K! cells (black) and after cholesterol oxidase treatment (blue); Figure S5: The plot of ABCA1 molecules number (N) in the function of the waist surface in A1G and MMG cell with or without ApoA1. The linear regression calculated for each data set correspond to the average diffusing molecules number (N) over different waists ± SE. A1G: 184.6 ± 34.13; MMG: 207.3 ± 23.65; A1G+ApoA1: 170.5 ± 20.05; MMG + ApoA1: 217.2 ± 46.61. The differences are not statistically significant.
