# Supplementary material for: Molecular Diffusion of ABCA1 at the Cell Surface of Living Cells Assessed by svFCS
Source: Membranes (Basel). 2021 Jun 30;11(7):498. doi: 10.3390/membranes11070498 (PMC8306713; doi:10.3390/membranes11070498)
Supplement: Supplementary file 1 [file membranes-11-00498-s001.zip › membranes-1235473-supplementary.pdf]

membranes

Communication

Molecular Diffusion of ABCA1 at the Cell Surface of Living Cells Assessed by svFCS

Olga Raducka-Jaszul [1], Karolina Wójtowicz [2], Aleksander F. Sikorski [3], Giovanna Chimini [4], Yannick Hamon [4] and Tomasz Trombik [5,*]

[1] Department of Cytobiochemistry, Faculty of Biotechnology, University of Wrocław, Joliot-Curie 14a, 50-383 Wrocław, Poland; olgaraducka@gmail.com
[2] Department of Biotransformation, Faculty of Biotechnology, University of Wrocław, Joliot-Curie 14a, 50-383 Wrocław, Poland; karolina.wojtowicz@uwr.edu.pl
[3] Research and Development Center, Regional Specialist Hospital, Kamieńskiego 73a, 51-154 Wroclaw, Poland; Sikorski.Aleksander@wssk.wroc.pl
[4] Aix Marseille University, CNRS, INSERM, CIML, Marseille, France; chimini@ciml.univ-mrs.fr (C.G); hamon@ciml.univ-mrs.fr (H.Y)
[5] Department of Biophysics, Faculty of Biotechnology, University of Wrocław, Joliot-Curie 14a, 50-383 Wrocław, Poland
* Correspondence: tomasz.trombik@uwr.edu.pl

Citation: Raducka-Jaszul, O.; Wójtowicz, K.; Sikorski, A.F.; Chimini, G.; Hamon, Y.; Trombik, T. Molecular Diffusion of ABCA1 at the Cell Surface of Living Cells Assessed by svFCS. *Membranes* **2021**, *11*, 498. https://doi.org/10.3390/membranes11070498

Academic Editor: Eva C. Arnspang

Received: 10 May 2021
Accepted: 29 June 2021
Published: 30 June 2021

Publisher's Note: MDPI stays neutral with regard to jurisdictional claims in published maps and institutional affiliations.

Copyright: © 2021 by the author. Licensee MDPI, Basel, Switzerland. This article is an open access article distributed under the terms and conditions of the Creative Commons Attribution (CC BY) license (http://creativecommons.org/licenses/by/4.0/).

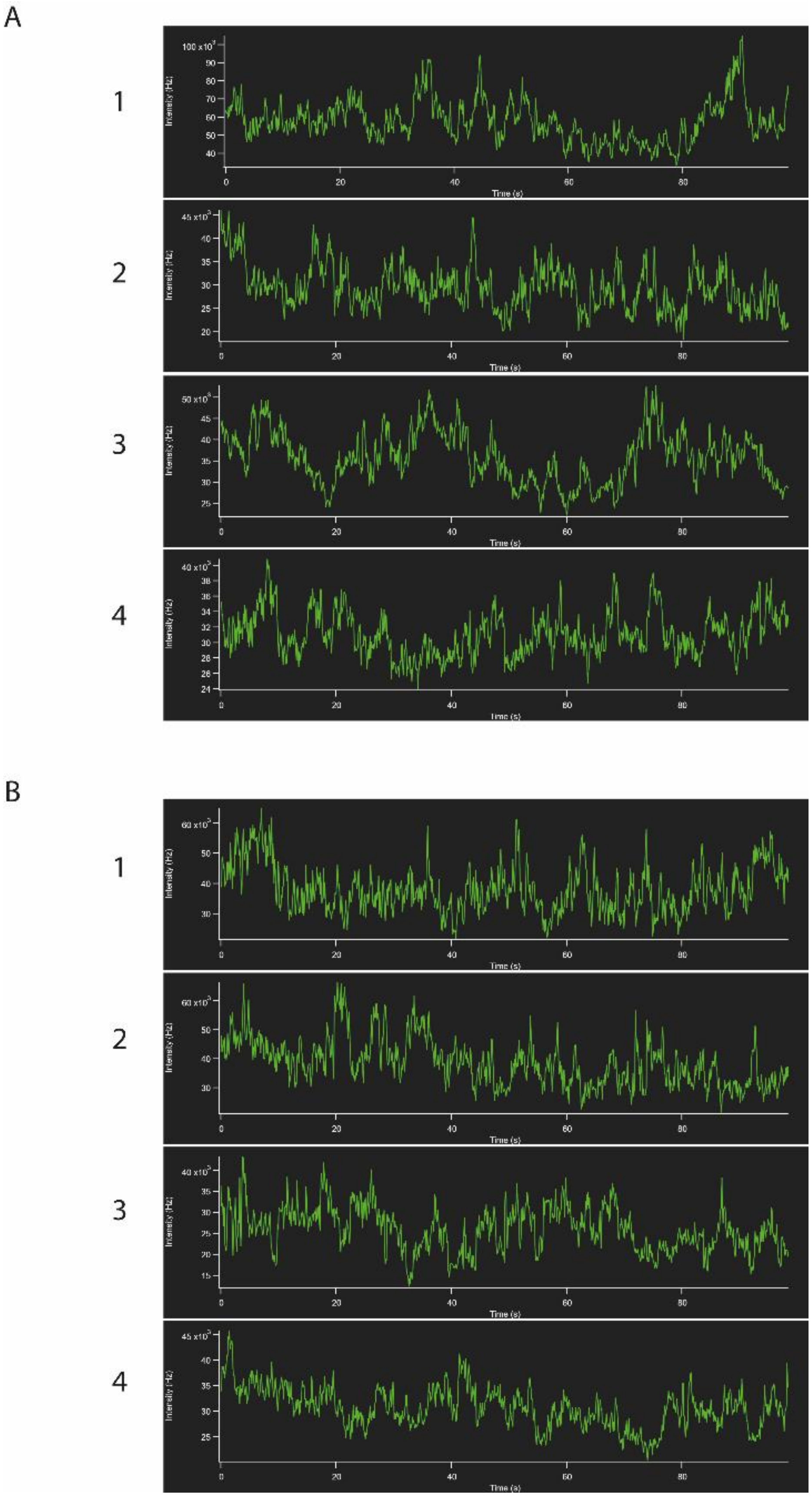

Figure S1. Representation of typical fluorescence fluctuation records from 100 s time frames for diffusion of ABCA1 in A1G cells (**A**), ABCA1MM in MMG cells (**B**) at different waist sizes (1: 0.15 μm^2, 2: 0.21 μm^2, 3: 0.27 μm^2, 4: 0.43 μm^2). A representation of fluorescence fluctuation record from 100 s time frame for the molecule which is photobleached is presented in C (waist size: 0.07 μm^2).

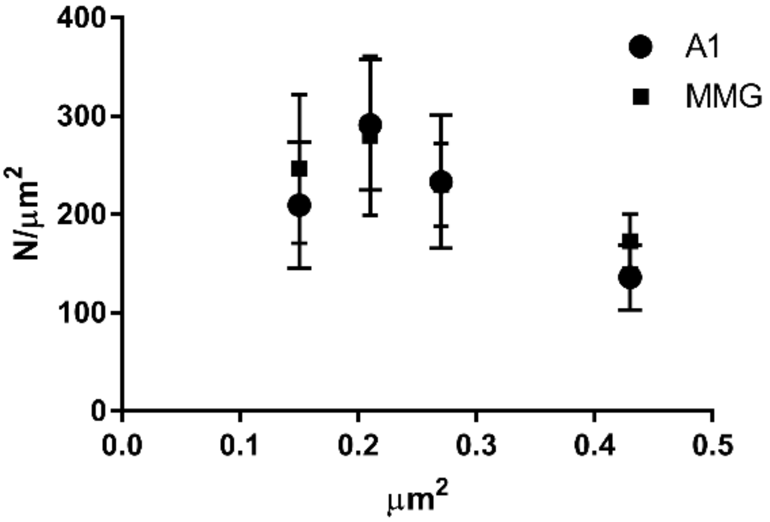

Figure S2. Evolution of ABCA1 molecule's density (N/μm²) in the function of the waist surface in A1G and MMG cells. The differences are not statistically significant.

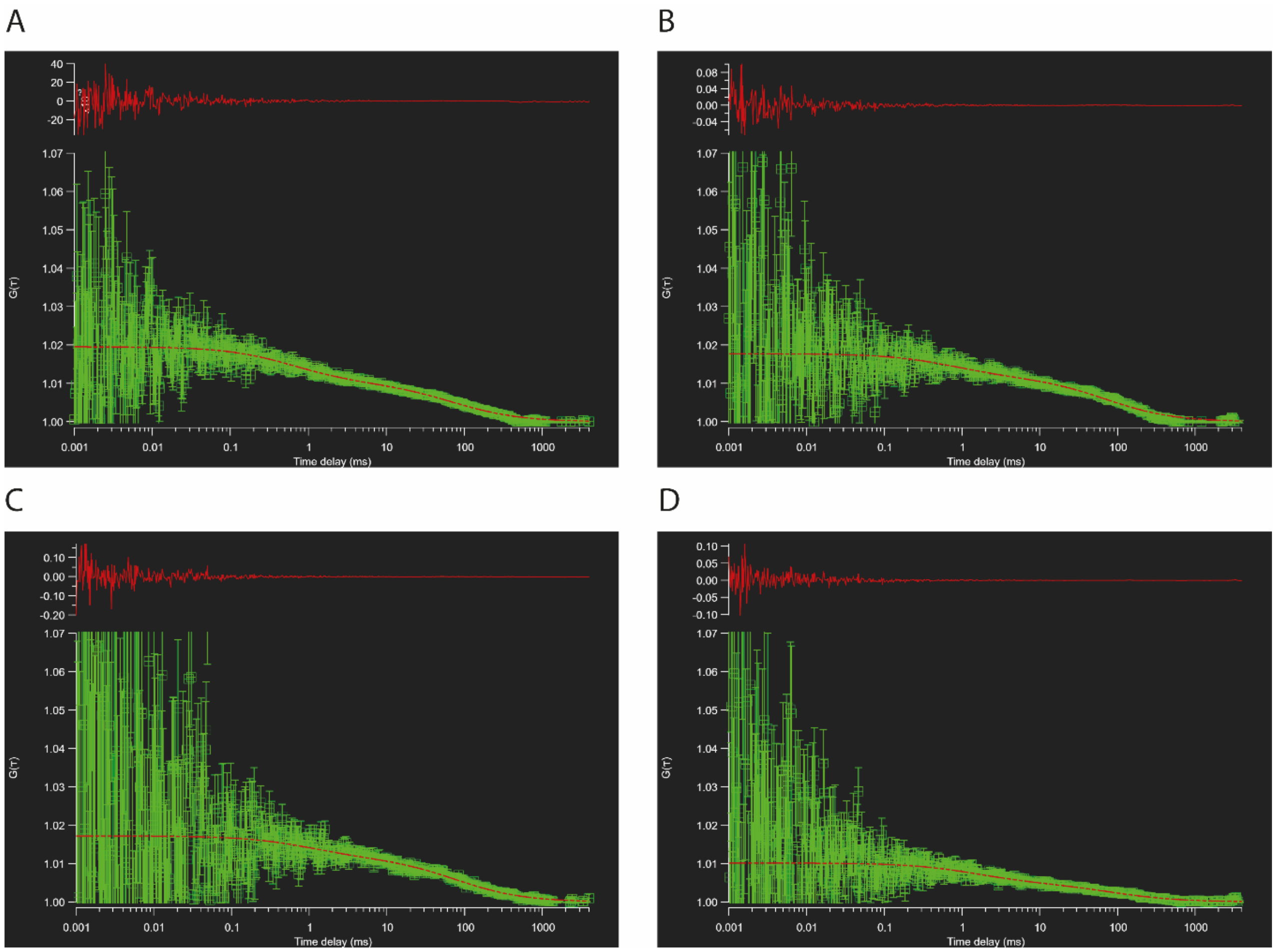

Figure S3. Examples of the multiple ACF fit (lower panel in green) with 2D diffusion model for ABCA1 in A1G cells at different waist surfaces (**A**: 0.15 μm², **B**: 0.21 μm², **C**: 0.27 μm², **D**: 0.43 μm²). The upper panel represents the deviation from the fit (in red).

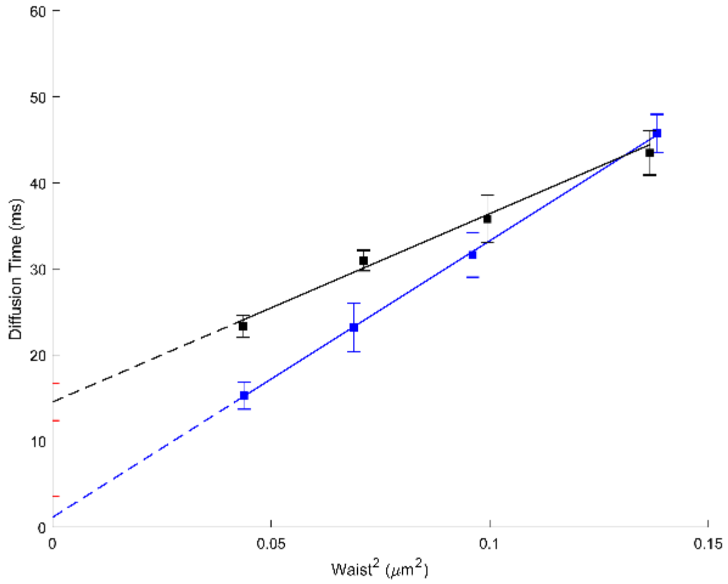

Figure S4. svFCS diffusion laws for a Bodipy-SM in non-treated CHO-K! cells (black) and after cholesterol oxidase treatment (blue).

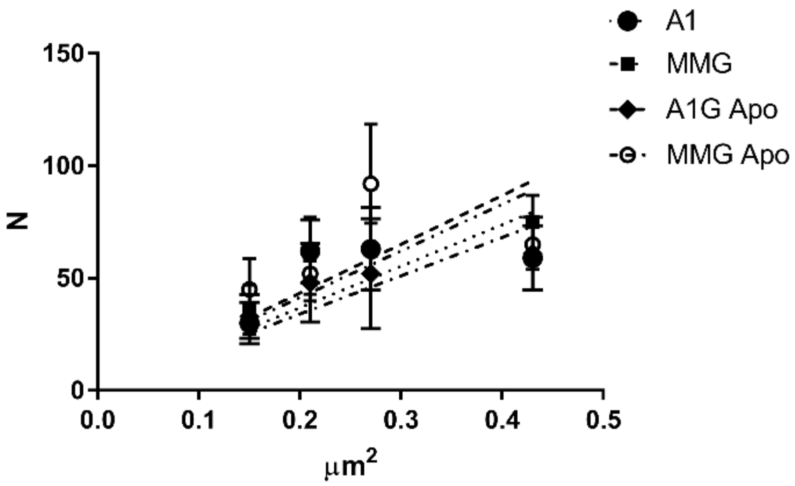

Figure S5. The plot of ABCA1 molecules number (N) in the function of the waist surface in A1G and MMG cell with or without ApoA1. The linear regression calculated for each data set correspond to the average diffusing molecules number (N) over different waists ± SE. A1G: 184.6 ± 34.13; MMG: 207.3 ± 23.65; A1G + ApoA1: 170.5 ± 20.05; MMG + ApoA1: 217.2 ± 46.61. The differences are not statistically significant. .
